# Electric stimulation of the medial forebrain bundle influences sensorimotor gaiting in humans

**DOI:** 10.1186/s12868-019-0503-y

**Published:** 2019-04-29

**Authors:** Patricia Panther, Maria Kuehne, Jürgen Voges, Sven Nullmeier, Jörn Kaufmann, Janet Hausmann, Daniel Bittner, Imke Galazky, Hans-Jochen Heinze, Andreas Kupsch, Tino Zaehle

**Affiliations:** 1 Department of Stereotactic Neurosurgery, University Hospital of Magdeburg, Magdeburg, Germany; 2 Department of Neurological Surgery, Ulm University Medical Center, Ulm, Germany; 3 Department of Neurology, University Hospital of Magdeburg, Leipziger Str. 44, 39120 Magdeburg, Germany; 4 Institute of Molecular and Cellular Anatomy, Ulm University, Ulm, Germany; 5 NEUROLOGY-MOVES, Academic Neurology Practice, Berlin, Germany; 6 Leibniz Institute for Neurobiology, Magdeburg, Germany

**Keywords:** Prepulse inhibition, PPI, Medial forebrain bundle, Deep brain stimulation, DBS, Reward system, Neuromodulation, Alzheimer’s disease

## Abstract

**Background:**

Prepulse inhibition (PPI) of the acoustic startle response, a measurement of sensorimotor gaiting, is modulated by monoaminergic, presumably dopaminergic neurotransmission. Disturbances of the dopaminergic system can cause deficient PPI as found in neuropsychiatric diseases. A target specific influence of deep brain stimulation (DBS) on PPI has been shown in animal models of neuropsychiatric disorders. In the present study, three patients with early dementia of Alzheimer type underwent DBS of the median forebrain bundle (MFB) in a compassionate use program to maintain cognitive abilities. This provided us the unique possibility to investigate the effects of different stimulation conditions of DBS of the MFB on PPI in humans.

**Results:**

Separate analysis of each patient consistently showed a frequency dependent pattern with a DBS-induced increase of PPI at 60 Hz and unchanged PPI at 20 or 130 Hz, as compared to sham stimulation.

**Conclusions:**

Our data demonstrate that electrical stimulation of the MFB modulates PPI in a frequency-dependent manner. PPI measurement could serve as a potential marker for optimization of DBS settings independent of the patient or the examiner.

## Background

Prepulse inhibition (PPI) of the acoustic startle response (ASR) is a physiological and operational measure of the pre-attentive filtering process known as sensorimotor gating [20]. PPI describes a reduction in the startle response amplitude, if an acoustic startling pulse is preceded by a non-startling stimulus (prepulse) at approximately 30 – 500 ms [12, 32]. The weak prepulse stimulus is thought to activate a pre-attentional gating mechanism that inhibits the startle response. Deficits in PPI can be found in several neuropsychiatric disorders like schizophrenia, obsessive compulsive disorders, Huntington’s and Parkinson’s disease (PD) [4, 5, 31, 36, 44]. Also dementia of Alzheimer type (AD) is discussed to diminish PPI [14, 27, 38]. Measurement of PPI is characterized by adequate face, predictive, and construct validity [35], but can be also modulated by attention and drugs [9, 17]. Additionally, PPI has been shown to be directly influenced by monoaminergic agents and is altered in diseases associated with dopaminergic dysfunction [5, 11]. Further, baseline PPI is suggested as an important determinant of the effect of dopamine agonists on PPI [1]. Although it does not require learning, the expression of PPI is regulated by higher cognitive processes [20]. A potential link between PPI expression and cognitive performance has been suggested, such that poor PPI may predict cognitive impairments [2]. With regard to the latter, patients with PD, which show higher levels of PPI, are reported to perform better on cognitive measures, attention and processing speed than patients with lower levels of PPI [44].

**Fig. 1:**
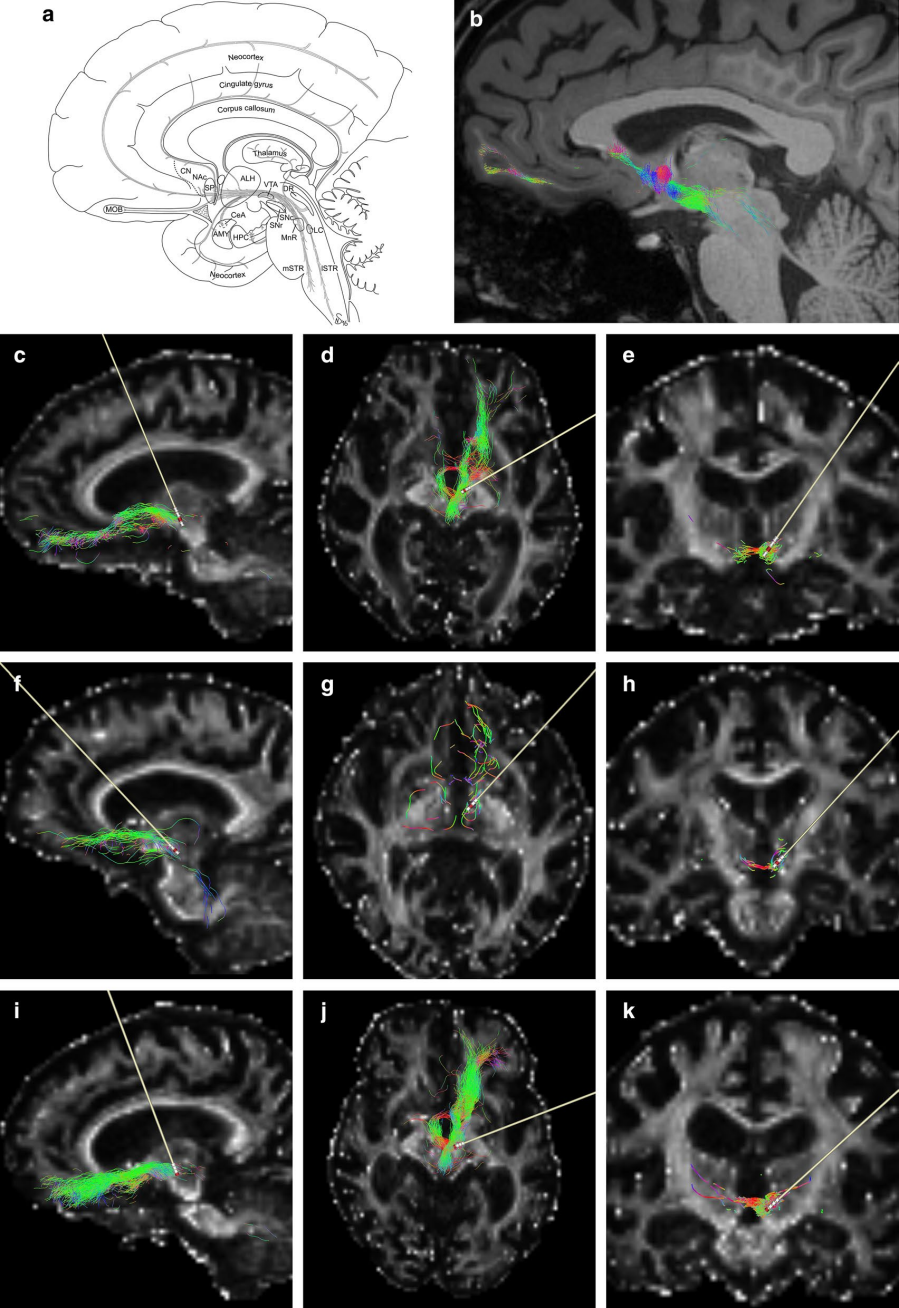
Schematical drawing and fiber reconstructions of the medial forebrain bundle. Schematical drawing (**a**) and fiber reconstruction based on diffusion imaging of a healthy control (**b**) illustrating the main projections of the fasciculus telencephali (medial forebrain bundle, MFB) are shown. Further, individual MFB reconstructions, with the corresponding electrode position for only the left side are shown for patient 1 (**c** sagittal-, **d** axial-, **e** coronal sections), patient 2 (**f**, **g**, **h**) and patient 3 (**i**, **j**, **k**). The activated contact is marked in red. The MFB consists of thin, loosely arranged ascending and descending fibers extending from septal area (SP) to the mesencephalic tegmentum. Along this route it traverses the lateral hypothalamic area (ALH) and splits into a smaller medial and larger lateral stream at transitional zone of diencephalon and midbrain. The medial stream (mSTR) passes through the parts of the mesencephalic and rhombencephalic tegmentum, connecting the hypothalamic centers with raphe nuclei and medial reticular formation. On the other hand, ascending serotonergic fibers from the dorsal (DR) and medial raphe (MnR) nuclei reach the ALH and a variety of diencephalic and telencephalic centers. The lateral stream (lSTR) connects the central nucleus of the amygdala (CeA) and hypothalamic areas with different brain stem areas in pons and medulla oblongata. It further comprises fibers ascending from dopaminergic ventral tegmental area (VTA) and substantia nigra pars compacta (SNc), but also fibers from noradrenergic fields like the locus coeruleus (LC) reaching cortical and limbic regions like hippocampus (HPC), amygdala (AMY) and nucleus accumbens (NAc). MOB—main olfactory bulb, SNr—substantia nigra pars reticulata, CN—cingulate gyrus

The median forebrain bundle (MFB) is a complex composition of monoaminergic fibre systems connecting midbrain and forebrain areas (Fig. 1a, b). It is involved in processing the dopamine dependent reward effect of electrical self-stimulation [42]. Dopaminergic modulation has been discussed as a therapeutic option to restore altered cortical plasticity in AD [19]. Conceivably, electrical stimulation of the MFB modulates dopaminergic pathways and could represent a potential therapeutic approach in AD, which we offered to three AD patients as compassionate use.

DBS allows focal and reversible neuromodulation. Data supports its efficacy in movement disorders, but also psychiatric diseases [15]. Finding the best stimulation setting in patients with a DBS-System can be challenging, especially if DBS does not exert rapid therapeutic response on the symptoms of the disease. Therefore, it is necessary to find tools indicating a change in the cerebral network activity which are easily and fast performed and react quickly on changes of the programming.

Since it was shown that pharmacologic manipulations of the dopaminergic systems alter sensorimotor gating [5, 11, 24, 33], we investigated if PPI can be influenced by DBS of the MFB in a stimulation frequency dependent manner, to elucidate its potential for optimisation of DBS setting.

## Methods

Ethical standards of Human and Animal Rights were adhered (including the Helsinki Declaration of 1975, as revised in 2000 and 2008) and the experimental design was approved by the local ethical committee (University of Magdeburg, Germany; reference numbers 07/12 and 131/13). In addition, the patients gave their informed consent for investigations addressing the influence of deep brain stimulation (DBS) of the MFB on PPI.

### Participants

Three patients suffering from mild Alzheimer’s disease (patient #1, female, age 79, Mini-Mental State Examination (MMSE): 27; patient #2, female, age 70, MMSE: 22; patient #3, male, age 70, MMSE: 17; all three patients were right handed) were offered bilateral DBS of the MFB (Fig. 1). This treatment option is not a standard, but a novel experimental approach in Alzheimer’s disease. Therefore DBS of the MFB was offered to these three patients as an individual treatment as compassionate use to improve cognition in AD. The patients were able to understand the experimental and pilot character of the method and the risk of the medical intervention and gave their written consent to participate in the study; continued ability to participate in the present study has been clinically verified by continued scientific compliance of the included patients. Furthermore, stimulation of MFB additionally offered the unique opportunity to scientifically explore the modulation of PPI by DBS, which was included in the written informed consent. Patients were tested in MMST to evaluate the severity of dementia. Additionally, to exclude unsystematic side effects during the postoperative stimulation period of 3–6 days, we assessed an extensive cognitive testing battery to ensure that our experimental manipulation did not harm the patients. The cognitive tests comprising visual learning, short delayed match to sample task, reward paradigms, Mini-Mental State Examination, Alzheimer’s Disease Assessment Scale Cognition, geriatric depression scale, tests for attentional performance, and spatial memory tests, all of them did not change significantly during the short postoperative stimulation period of 3–6 days. Finally, to exclude the possibility of a hearing loss all patients were neurologically examined. Patients were recruited from the Departments of Neurology and Stereotactic Neurosurgery at the University Hospital of Magdeburg (for details cf. Table 1). Clinical diagnosis was confirmed by D. B.

**Table 1:** Patient characteristics

	Patient #1	Patient #2	Patient #3
Disease duration [years]	6	5	6
Medication [years]	Donepezile 10 mg [3] Donepezile 5 mg [1] Gingko 240 mg [0.25] Mirtazapine 30 mg [3]	Rivastigmine 9.5 mg [2] Citalopram 20 mg [0.75]	Rivastigmine 9.5 mg [2] Gingko 240 mg [0.5]
Severity of dementia	Mild AD, MMSE 27	Mild AD, MMSE 22	Moderate AD, MMSE 17
Clinical course	Slowly progressive with an increase of progression over the past year	Slowly progressive	Slowly progressive
Cognitive deficits	Encoding/consolidation memory, spatial and temporal orientation, executive functions	Encoding/consolidation memory, spatial and temporal orientation, visoconstructional skills	Encoding/consolidation memory, spatial and temporal orientation, visoconstructional skills, language, apraxia
MRI	Mild bilateral hippocampal atrophy	Moderate bitemporal atrophy including hippocampal atrophy	Global cortical atrophy
CSF	Amyloid beta ↓ Phosphorylated tau levels ↑	Amyloid beta ↓ Phosphorylated tau levels ↑	Amyloid beta ↓ Phosphorylated tau levels →

### Surgery and electrical stimulation

Surgery was performed as previously described [40]. The MFB was localized using tractography and the known anatomic relationship to the ventral tegmental area (VTA) and subthalamic nucleus (STN) as elaborated by Coenen et al. [6]. Brain electrodes (Model 3389, Medtronic^®^, Minneapolis, MN, USA) were placed bilaterally in the superolateral branch of the MFB on both sides under general anesthesia and connected to an impulse generator (Activa-PC^®^, Medtronic^®^). Intraoperative stereotactic X-rays and postoperative CT images documenting the electrode position were fused with the preoperative (i.e. “planning”) MRI and tractography using Praezis-Plus^®^ planning software (Precisis AG, Walldorf, Germany) in order to confirm the localization of stimulation electrodes relative to the MFB (Fig. 1 and Table 2). Prior to PPI measurement the patients were treated in 12-h intervals according to a standardized protocol: sham-stimulation followed by continuous stimulation with 20 Hz, 60 Hz or 130 Hz in a pseudorandomized order. The pulse width was set at 90 µs and below the occurrence of side effects (e.g. unrest, sweating, widening of the pupils, oculomotor distortion). The stimulation voltage was increased in steps of 0.2 V every 20 min to analyse possible side effects. Depending on the stimulation frequency, adverse effects occurred at 20 Hz by 2.8–3.4 V, 60 Hz at 1.9–2.8 V and 130 Hz at 1.6–2.2 V. If side effects occurred, the voltage was reduced by steps of 0.1 V until they disappeared. An individual voltage (see Table 2) was chosen according to appearance of side effects. Patients showed a stable condition without complications using a stimulation frequency at 60 Hz. Data from the follow up will be provided in another manuscript.

**Table 2:** Stimulation parameters

	Patient #1		Patient #2		Patient #3	
	Right electrode	Left electrode	Right electrode	Left electrode	Right electrode	Left electrode
ACPC-length (mm)	24.8		22.4		27.0	
Right–left (mm)	+ 6.8	− 7.7	6.9	− 4.6	5.8	− 5
Anterior–posterior (mm)	− 3.4	− 3.4	− 1.2	± 0	− 4.5	− 4.5
Dorsal ventral (mm)	− 4.7	− 6.5	− 4.9	− 4.2	− 4.6	− 5.1
Active contacts (mm)	8–, G+	1–, G+	10–, G+	1–, G+	9–, G+	0–, G+
Voltage (V)	2	2	2	1.6	1.5	2

### PPI data acquisition

All participants were examined in a quiet room, while sitting in an armchair with the knees flexed, and were asked to remain awake and relaxed. After detection of bilateral hearing thresholds, acoustic stimuli were binaurally presented through headphones. Each startle session comprised a modulatory prepulse stimulus (80 dB SPL at 1000 Hz; rise/fall time 5 ms, 30 ms duration), which preceded a startle stimulus (pulse-alone; 100 dB SPL at 1000 Hz; rise/fall time 5 ms, 30 ms burst of pure tone) by 30, 60 or 120 ms (prepulse–pulse). The acoustic startle session started with a 1 min acclimation period followed by 4 initial pulse-alone trials for acclimation. These trials were not included into the analysis. Afterwards, the acquisition period was performed with 15 pulse-alone trials and 45 prepulse–pulse trials, presented in a pseudorandomized order. The inter-trial intervals varied between 8 and 22 s. A 65 dB SPL broadband noise (0–44 kHz) was presented as a background noise throughout the session that lasted approximately 20 min.

The ASR recordings were carried out at days 3–6 post-surgery, starting with the sham stimulation followed by three separate verum stimulation conditions (20 Hz, 60 Hz, 130 Hz) at four consecutive days. PPI sessions were performed in the morning after 12 h of continuous stimulation at each specific stimulation frequency or no stimulation (sham condition). The stimulation duration of 12 h was chosen to avoid initiation effects or a possible latency period.

Both, patients and the examiner were blinded about the current stimulation frequency (double-blind-design). The eye-blink component of the ASR was measured by electromyography (EMG) recordings from pure-tin electrodes (1 cm diameter) placed below the left eye at the inferior orbicularis oculi and at the outer canthus (lateral orbicularis oculi muscle). EMG data were acquired continuously by using a BrainAmp amplifier system (BrainProducts, Gilching, Germany) with a sampling rate set at 500 Hz, and signals band-limited to 250 Hz. Electrode impedance was lower than 5 kΩ. Startle reflex were off-line analyzed using BrainVision Analyzer 2 (BrainProducts, Gilching, Germany). The EMG raw signal was off-line band-pass filtered between 0.5 and 20 Hz (slope 12 dB/octave) and then epoched based on the stimulus onset with 150 ms preceding stimulus onset and 300 ms of data poststimulus. After the segmentation the data were baseline corrected by using a 50 ms pre-stimulus interval. Each EMG response was visually inspected for artefact rejection. Voluntary and spontaneous blinks were excluded from further analysis. With a moving average of 50 ms the data were smoothed. Response peak was defined as the point of maximal amplitude that occurred within a window of 20–250 ms after stimulus onset. In order to account for individual differences in startle amplitude [24] prepulse inhibition was assessed as the percentage of reduction of the amplitude after pulse-alone trials [i.e., PPI = (PA − PP)/PA × 100], where PA indicates amplitude after pulse-alone trials and PP indicates the amplitude after prepulse–pulse trials.

### Data acquisition of patient scans

The scans were performed to verify the anatomical structure of MFB for stereotactic surgery individually (Fig. 1). The MRI scans were performed on a Siemens Verio 3T system (Siemens Medical Systems, Erlangen, Germany) equipped with a gradient coil capable of 45 mT/m and 200 T/m/s slew rate. A standard 32-channel phased array imaging coil was used. To increase inter-subject reproducibility in position and minimize motion a thin pillow was placed surrounding the sides and the back of the head. The field of view was aligned in all cases to the anterior commissure–posterior commissure (ac–pc) line.

Diffusion images were acquired using a twice refocused, single shot, echo planar imaging pulse sequence using the following parameters: TE/TR = 86/10,400 ms, matrix size = 128 × 128; 72 contiguous slices, yielding an isotropic resolution of 2 × 2 × 2 mm^3^, receiver bandwidth of 1698 Hz/pixel and an echo spacing of 0.69 ms. Diffusion weighted images were acquired along 20 non-collinear diffusion directions with b = 1000 s/mm^2^ and one scan without diffusion weighting (b = 0 s/mm^2^) and two averages. We allowed for parallel acquisition of independently reconstructed images using generalized auto calibrating, partially-parallel acquisitions or GRAPPA [13], with acceleration factor of 3 and 57 reference lines. The total acquisition time was 8 min 09 s. T1-weighted high resolution structural MRI images were obtained using a 3D-MP RAGE sequence with the following parameters: TE/TR = 7.21/2700 ms, TI = 1100 ms, flip angle = 7°, receiver bandwidth = 130 Hz/pixel and a matrix size of 256 × 256 × 176, yielding to an isotropic resolution of 1 mm^3^. The total acquisition time was 7 min 34 s.

### Data acquisition of volunteer MRI scan

MRI scans of healthy volunteers were made to demonstrate the regular anatomy and reproducibility of the MFB (n: 11, 6♂, 5♀, age: 29 ± 5 years). The volunteer MRI scan was performed on a Siemens Prisma 3T system (Siemens Medical Systems, Erlangen, Germany) equipped with a gradient coil capable of 80 mT/m and 200 T/m/s slew rate. A standard 64-channel phased array imaging coil was used in receive mode.

Diffusion tensor images were acquired using a monopolar diffusion encoding, single shot, echo planar imaging pulse sequence using the following parameters: TE/TR = 49/10,200 ms, matrix size = 138 × 138; 90 contiguous slices, yielding an isotropic resolution of 1.6 × 1.6 × 1.6 mm^3^, receiver bandwidth of 2012 Hz/pixel and an echo spacing of 0.62 ms. Diffusion weighted images were acquired along 60 non-collinear diffusion directions with b = 1000 s/mm^2^ and 13 scans without diffusion weighting (b = 0 s/mm^2^) equidistant distributed between the diffusion weighted scans. In order to correct for eddy current-induced distortions for each gradient orientation, diffusion-weighted measurements were acquired with both gradient polarities [3] adding up to a total of 120 diffusion-weighted volumes. We allowed for parallel acquisition of independently reconstructed images using generalized auto calibrating, partially-parallel acquisitions or GRAPPA [13] with acceleration factor of 3 and 36 reference lines. The total acquisition time was 23 min 38 s.

T1-weighted high resolution structural MRI images were obtained using a 3D-MP RAGE sequence with the following parameters: TE/TR = 4.50/2600 ms, TI = 1100 ms, flip angle = 7°, receiver bandwidth = 140 Hz/pixel and a matrix size of 320 × 320 × 240, yielding to an isotropic resolution of 0.8 mm^3^. The total acquisition time is 13 min 52 s.

### Data processing and fiber tracking

Using the MP RAGE scan anatomical regions were manually delineated for the nucleus accumbens and the VTA. Based on FreeSurfer segmentation (version 5.3.0; https://surfer.nmr.mgh.harvard.edu/) [7], the prefrontal cortex was automatically segmented and binary masks were created using a home build MATLAB (Mathworks^®^, Natick, MA, USA) script. All binary masks were coregistered into the diffusion space using FSL (version 5.0.9, http://fsl.fmrib.ox.ac.uk/fsl/fslwiki/) flirt and fnirt tools [16]. Diffusion data were eddy current and motion corrected using the FSL tools eddy_correct and flirt. Diffusion gradient directions were corrected according to detected head motions. Using the MRtrix package (https://github.com/MRtrix3/mrtrix3), [37] constrained spherical deconvolution (CSD) provide fiber orientation distribution function which were used for probabilistic tractography. For each start region in the VTA bilateral binary mask 10^7^ fibers were started and filtered ipsilateral with a waypoint region in the nucleus accumbens and a target region in the prefrontal cortex. The resulting representative pathways were visualized for the left hemisphere using the mrview tool of the MRtrix package as overlay to fractional anisotropy maps.

### Statistics

Since Kolmogorov–Smirnov tests indicated violations of the normality assumption, for each patient, PPI indices were analysed using non-parametric Friedman tests with the factor DBS (Sham, STIM I, STIM II, STIM III). Subsequently planned comparisons for each patient between verum and sham stimulation conditions by means of non-parametric Wilcoxon signed-rank tests (one-tailed) were performed and Bonferroni-Holms corrected for multiple comparisons. Data are presented as mean ± SEM (standard error of the mean).

## Results

**Fig. 2:**
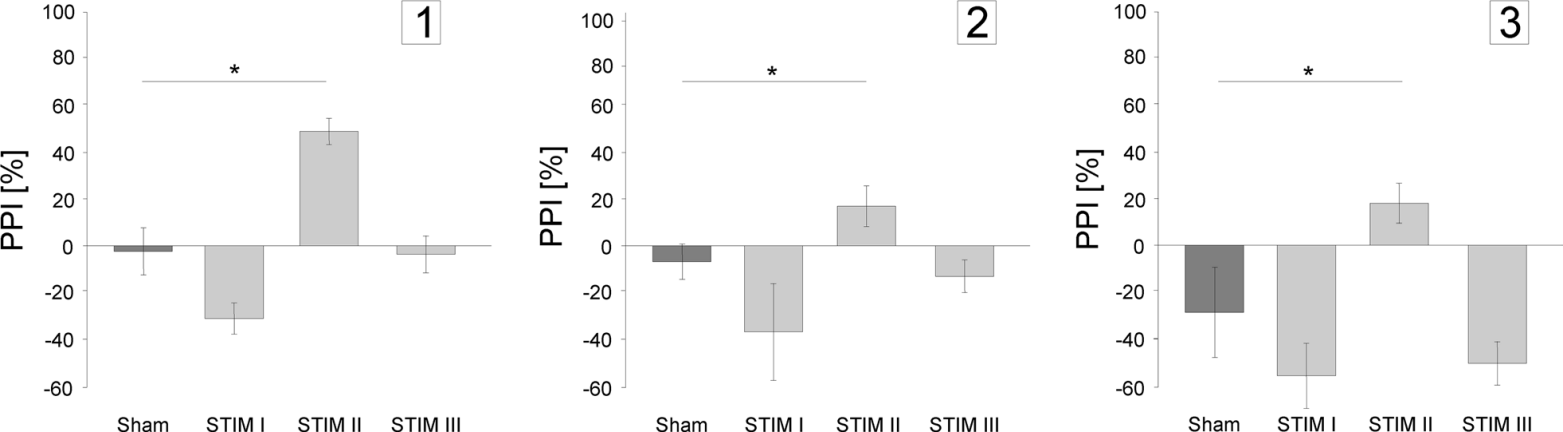
Frequency dependent effects of MFB stimulation on prepulse inhibition of acoustic startle response (PPI): PPI of all three patients (#1–3) is significantly increased following 60 Hz stimulation compared to Sham-stimulation (Wilcoxon test: *p < .05)

In order to assess the impact of MFB–DBS on PPI in three single cases of AD, we compared PPI under sham stimulation with PPI during three different DBS stimulation settings separately for all each patients (Sham; STIM I: 20 Hz; STIM II: 60 Hz; STIM III: 130 Hz). As shown in Fig. 2, DBS systematically modulated the PPI in each patient [Patient #1: DBS χ^2^(3) = 28.55, *p* < .001; Patient #2: χ^2^(3) = 8.169, *p* < .05; Patient #3: χ^2^(3) = 24.20, *p* < .001]. In all three patients the PPI was significantly enhanced during STIM II (60 Hz) (Patient #1: z = −3.385, *p* < .003; Patient #2: z = −2.53, *p* = .04; Patient #3: z = −2.67, *p* = .012) compared to Sham, while PPI was unaffected in STIM I (20 Hz) (Patient #1:z = −1.73, *p* = .08; Patient #2: z = −0.57, *p* = .27; Patient #3: z = −1.36, *p* = .09) and STIM III (130 Hz) condition (Patient #1: z = −0.17, *p* = .43; Patient #2: z = −0.74, *p* = .18; Patient #3: z = −1.7, *p* = .09). In summary, compared to sham-stimulation, 60 Hz DBS of the MFB increased PPI consistently in all three patients.

## Discussion

Three patients with AD received DBS of the MFB. This offered us the rare opportunity to investigate the influence of MFB–DBS on sensorimotor gating. To our knowledge, the present study describes for the first time the effects of MFB–DBS on prepulse inhibition of acoustic startle reflex (PPI) in humans. We show that short-term MFB–DBS modulates PPI in a frequency dependent manner with 60 Hz significantly enhancing PPI in all patients while 20 Hz and 130 Hz did not affect PPI.

The post-experimental chronic stimulation frequency was set to 60 Hz for all three patients. This decision was not only based on the experimental data reported in the present manuscript, but also on the absence of unwanted side effects at low voltages, which occurred during 130 Hz stimulation (unrest, increased sweating and widening of the pupils). Additionally, at 60 Hz continued stable clinical cognition parameters were observed.

The effect that an intermediate stimulation frequency of 60 Hz improves PPI in contrast to low or high stimulation frequencies can be described by a bell shaped function (see Fig. 2).

Although the present investigation did not address the specific mechanisms of DBS, it is possible to speculate that the neurotransmitter dopamine [18], could be involved in the frequency dependent alteration of PPI following MFB–DBS.

Previous experimental studies on the effects of electrical stimulation of the MFB demonstrated that a stimulation frequency of 60 Hz is able to increase dopamine release in VTA, caudate putamen, nucleus accumbens and medial prefrontal cortex of rodents [10, 18, 26]. The dopamine overflow in striatum during MFB stimulation showed a frequency dependent, bell shaped pattern, with a maximum at 60–100 Hz in rodents and at 80–100 Hz in non-human primates followed by a decrease at higher stimulation frequencies [8, 30]. Additionally, frequency dependent bell shaped effects of the STN–DBS on striatal dopamine release have been reported in rodents with a maximal peak effects occurring at 50 Hz frequency [22]. Furthermore, positive correlation between PPI and dopamine transporter ligand ^123^I-FP-CIT uptake in the striatum in patients with PD disease was shown [44].

Studies investigating DBS in PD indicate that beside the precision of implantation, treatment efficacy depends on the frequency of stimulation [41]. For example, STN–DBS at 130 Hz improves medication refractory tremor, whereas STN–DBS at 60 Hz improves especially freezing of gait [25]. However, also only immediate positive effects (at 80 Hz; [29] or the absence of a benefit at 60 Hz compared to 130 Hz DBS on freezing of gait have been reported [28, 39]. Stimulation at 20 Hz is known to deteriorate motor symptoms, but is able to increase verbal fluency at 10 Hz [43]. Dopamine is able to disturb PPI in higher dosages [34]. Therefore, it is assumed that stimulation frequency differentially influences dopamine release and this could lead to a change in prepulse inhibition in different directions. Depending on this, a low frequency (20 Hz), intermediate (60 Hz) and a high frequency (130 Hz) was chosen to test PPI in MFB–DBS. However, it should be noted that frequency dependent dose–response curves have also been observed for extracellular serotonin in the caudate nucleus of rats following MFB-stimulation [23]. Considering this, it remains unclear which neurotransmitter systems are modulated by DBS of the MFB. Finally, Kohl et al. [21] recently showed that PPI is normalized following DBS in nucleus accumbens in patients with obsessive compulsive disorders. However, no frequency dependence was investigated in their study [21]. Independently, the latter and the present study demonstrate that PPI may represent a surrogate marker for network communication in neuropsychiatric diseases, which can be modulated by DBS.

## Conclusion

In summary, we found that MFB–DBS in AD patients modulates PPI in a frequency-dependent manner. A concomitant increase of PPI was observed at 60 Hz stimulation, while deteriorated or unchanged PPI effects were seen at 20 and 130 Hz. Intriguingly, similar bell shaped frequency effects have been observed in animal studies investigating striatal dopamine release during MFB stimulation [8, 30]. With respect to, that an increased PPI associated with better strategy formation and execution times in healthy males [2], it is unclear why an increase of PPI in 60 Hz in AD is not accompanied with improvement in cognition. PPI is mediated by a complex circuit influenced by various subcortical and cortical brain regions and neurotransmitter systems [20]. As a consequence it is possible that DBS of the MFB improves PPI independently from cognition. Further studies are necessary to investigate different stimulation modes or longer stimulation periods (chronic stimulation) and their influences on PPI. Taking into account the disease- and target specificities, these approaches may identify PPI as a potential marker for optimization of DBS settings independent of the patient or the examiner and in disease entities with delayed responding times for clinical symptoms following DBS.

## Abbreviations

ASR: acoustic startle response
AD: alzheimer dementia
AMY: Amygdala
CSD: constrained spherical deconvolution
DBS: deep brain stimulation
EMG: electromyography
MFB: median forebrain bundle
MMSE: mini-mental state examination
PD: Parkinson’s disease
PPI: prepulse inhibition
STN: subthalamic nucleus
VTA: ventral tegmental area
